# Nutritional diversity and bioactive potential of thirteen wild edible insects in India: implications for sustainable food and nutrition security

**DOI:** 10.3389/fnut.2026.1942254

**Published:** 2026-10-14

**Authors:** Lobeno Mozhui, L. N. Kakati, Neha Trivedi, Aditya Kumar Tanwar, Mukesh Kumar Dhillon, Bendang Ao, Pranay Punj Pankaj, Rita Khatri Chhetri, Jasha Momo H. Anal

**Affiliations:** 1 Department of Zoology, Nagaland University, Lumami, India; 2 Faculty of Science, Assam down town University, Guwahati, Assam, India; 3 Department of Biochemistry, College of Basic Science and Humanities, Govind Ballabh Pant University of Agriculture & Technology, Pantnagar, Uttarakhand, India; 4 Division of Entomology, IARI, New Delhi, India; 5 Natural Products and Medicinal Chemistry Division, CSIR- Indian Institute of Integrative Medicine, Jammu, India; 6 Academy of Scientific and Innovative Research (AcSIR), Ghaziabad, India

**Keywords:** amino acids, edible insects, fatty acids, functional foods, nutritional composition, traditional food

## Abstract

**Background:**

Edible insects are increasingly recognized as sustainable alternative food resources owing to their high nutritional value, diverse bioactive compounds, and low environmental footprint. However, comprehensive nutritional characterization of many wild edible insect species remains limited. Therefore, this study evaluated the nutritional composition, amino acid and fatty acid profiles, bioactive properties, and multivariate nutritional relationships of 13 wild-harvested edible insect species traditionally consumed in Nagaland, India.

**Methods:**

Proximate composition, total phenolic content, antioxidant content, amino acid composition, and fatty acid profiles were determined via standard analytical methods. Spearman’s correlation analysis, principal component analysis (PCA), and chord diagram visualization were employed to examine associations among nutritional variables and species-specific nutritional characteristics.

**Results:**

Across all 13 species, protein content ranged from 18.90 to 63.91%, while the lipid content varied from 4.31 to 35.08%. The total phenolic content ranged from 2.80 to 13.89 mg GAE g^−1^, and the antioxidant content ranged from 2.01 to 45.11 mg AAE g^−1^. Eight essential and nine nonessential amino acids were detected across all species, with leucine and proline as the predominant amino acids. Fatty acid profiling revealed myristic, palmitic, stearic, palmitoleic, oleic, and linoleic acids as the major fatty acids, with monounsaturated fatty acids constituting the dominant lipid fraction (46.28–56.90 μg 100 g^−1^), followed by saturated (29.69–42.50 μg100 g^−1^) and polyunsaturated fatty acids (0.11–20.12 μg 100 g^−1^). Strong positive Spearman’s correlations were observed between carbohydrate and phenolic content (*ρ* = 0.782) and carbohydrate and antioxidant content (*ρ* = 0.784). Phenolic and antioxidant contents were also positively correlated (*ρ* = 0.629). Multivariate analyses, including principal component analysis and chord diagram visualization, revealed distinct species-specific nutritional profiles. *Provespa barthelemyi* larvae presented relatively high concentrations of essential and nonessential amino acids and oleic acid. In contrast, *Oecophylla smaragdina* and *Omphisa fuscidentalis* were more strongly associated with elevated phenolic and antioxidant contents.

**Conclusion:**

Our findings demonstrate significant interspecific variation in proximate composition, amino acid and fatty acid profiles, phenolic and antioxidant content, underscoring the importance of species-specific nutritional characterization when selecting edible insects for nutritional enrichment in value-added product development and their potential contribution to sustainable food systems and future food security.

## 1 Introduction

Global food systems face increasing pressure from population growth, environmental degradation, and climate variability, necessitating sustainable, nutrient-dense alternative food sources. Despite sustained global efforts, various forms of malnutrition, including undernutrition (micronutrient deficiency), overnutrition (obesity), and protein–energy malnutrition (PEM), continue to pose a significant threat to human health and development (1). These challenges are further exacerbated by emerging global crises that disrupt food systems and underscore the urgent need for sustainable, resilient solutions. The COVID-19 pandemic has underscored the global lack of preparedness for unforeseen disruptions to food supply chains and nutritional security, highlighting the urgent need for innovative strategies to ensure consistent and equitable access to food (2).

Conventional agricultural expansion, driven by rapid population growth and increasing food demand, has become a major contributor to environmental degradation, threatening approximately 86% of the species most vulnerable to extinction (3). Simultaneously, climate change, extreme weather events, global supply chain disruptions, and geopolitical uncertainties continue to increase the vulnerability of conventional food systems and intensify food insecurity. The growing demand for animal-derived protein, coupled with declining agricultural land and the substantial environmental footprint of livestock production, has further challenged the sustainability of traditional agriculture and macro-livestock systems (4). In response, several strategies, including dietary supplementation, food fortification, and dietary diversification, have been proposed to address malnutrition. Among these, dietary diversification is considered the most sustainable and culturally acceptable approach, promoting the use of diverse, nutrient-rich foods from local and traditional food systems (5). Although plant-based diets and cultured meat alternatives represent important advances toward sustainable food systems, their adoption may be constrained by economic, cultural, and regional factors, highlighting the need to identify additional sustainable nutrient sources to support future food and nutritional security (6).

Among the alternative food resources receiving increasing attention, edible insects have emerged as a promising solution to address global food and nutritional challenges. They are traditionally consumed across Asia, Africa, and Latin America and are rich in high-quality protein, essential amino acids, beneficial fatty acids, and micronutrients. In many rural and indigenous communities, entomophagy contributes to dietary diversification and nutritional security while supporting livelihoods and the sustainable use of biodiversity (7–9). Their potential has been increasingly recognized through international initiatives and global assessments promoting edible insects as sustainable food resources (10–16). However, their wider adoption remains limited by insufficient nutritional data, the lack of standardized evaluation, cultural perceptions, and regulatory challenges.

Nagaland is one of India’s richest regions for edible insect diversity, with 122 edible insect species (Supplementary Table S1) and 59 therapeutic insect species (Supplementary Table S2) documented to date, alongside extensive indigenous knowledge of entomophagy and entomotherapy (15, 17–21). This represents a significant advancement in understanding the entomofaunal diversity and utilization of insects in Nagaland, positioning the present study among the most extensive regional assessments in this domain. The taxonomic distribution of the documented species reflects considerable diversity, with Hymenoptera accounting for the largest proportion (25%), followed by Hemiptera (21%), Orthoptera (20%), Coleoptera (15%), Lepidoptera (7%), and Odonata (6%), while Mantodea and other minor orders such as Megaloptera, Plecoptera, Blattodea, and Diptera collectively constitute the remaining fraction (Figure 1). Notably, several species have been newly recorded in the edible insect inventory of Nagaland, including *Pycanum* sp., *Cryptotympana corvus*, *Apriona germari*, *Neoplocaederus obesus*, *Tetragonula iridipennis*, *Tetragonula laeviceps*, *Tetragonula gressitti*, and *Lepidotrigona ventralis*. The inclusion of these species expands the known spectrum of edible insects in the region and underscores the richness of indigenous knowledge systems that continue to identify and utilize diverse bioresources.

**Figure 1:**
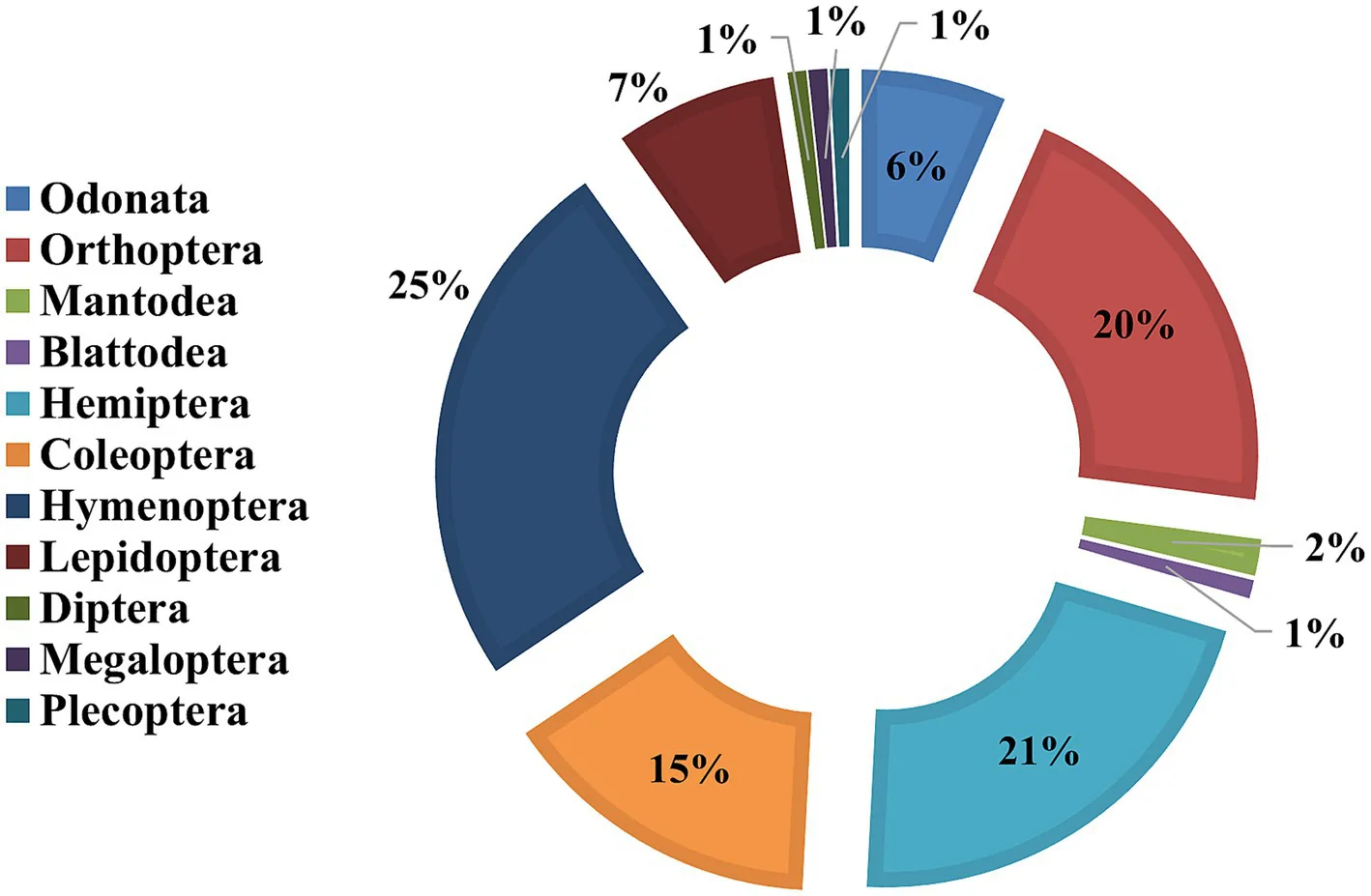
Distribution of edible insect species across different insect orders.

Ethnographic observations revealed a remarkable diversity of traditional culinary practices for preparing edible insects in Nagaland. These practices include boiling in a small amount of water, frying in hot oil, cooking with locally available ingredients, roasting over an open fire or charcoal, steaming, consuming them raw, and preparing them as chutney. Despite the considerable diversity of edible insects and the extensive traditional knowledge surrounding their consumption, significant research gaps remain in Nagaland. Nutritional information is available for only a limited number of edible insect species, while several traditionally consumed species remain poorly characterized. Existing studies have largely focused on individual species or selected nutritional components, limiting comprehensive comparative assessment of their nutritional value. Moreover, data on bioactive properties, particularly phenolic compounds and antioxidant potential, remain scarce for many edible insects from the region. Nutritional composition also varies considerably among species, developmental stages, diets, and habitats (22–25), making comparisons across existing studies difficult and highlighting the need for standardized and comparative evaluation. In addition, although traditional processing is an integral part of insect consumption in Nagaland, its potential influence on nutritional and bioactive properties has received limited systematic attention. Consequently, a clear gap exists between the extensive ethnobiological knowledge of edible insects in Nagaland and the scientific evidence required to establish their nutritional and functional potential. Addressing these gaps is essential for assessing their suitability as sustainable alternative food resources and their potential contribution to food and nutritional security. Therefore, this study comparatively evaluated the nutritional composition and bioactive properties of 13 representative edible insect species from Nagaland to assess their potential as sustainable alternative food resources.

## 2 Materials and methods

### 2.1 Sample collection and preparation

Thirteen edible insect species, namely *Coridius singhalanus*, *Cossus* sp., *Cybister limbatus*, Dragonfly nymphs, *Hydrophilus caschmirensis*, *Laccotrephes ruber*, *Lethocerus indicus*, *Mecopoda elongata*, *Omphisa fuscidentalis*, *Oecophylla smaragdina*, *Provespa barthelemyi*, *Samia cynthia ricini,* and *Tarbinskiellus portentosus,* were collected from their natural habitats of Nagaland via standard methods such as handpicking, sweep-netting, and aquatic sampling as per the ecological niche of each species. Representative specimens of the 13 edible insect species included in the present study are presented in Figure 2, providing a visual representation of the taxa collected. Collections were performed during appropriate seasons and times of peak activity to ensure the availability of the species (15). The samples were brought to the Ecology Laboratory, Department of Zoology, and processed for biochemical tests. For each insect species/developmental stage, multiple individuals were collected and pooled prior to sample preparation. Both male and female individuals were present in the collected samples and were included in the pooled material. The samples were then washed thoroughly with tap water followed by distilled water, oven-dried at 50 ± 5 °C for 48–72 h, ground into a fine homogeneous powder, and stored in sterile airtight containers at −80 °C until biochemical analyses.

**Figure 2:**
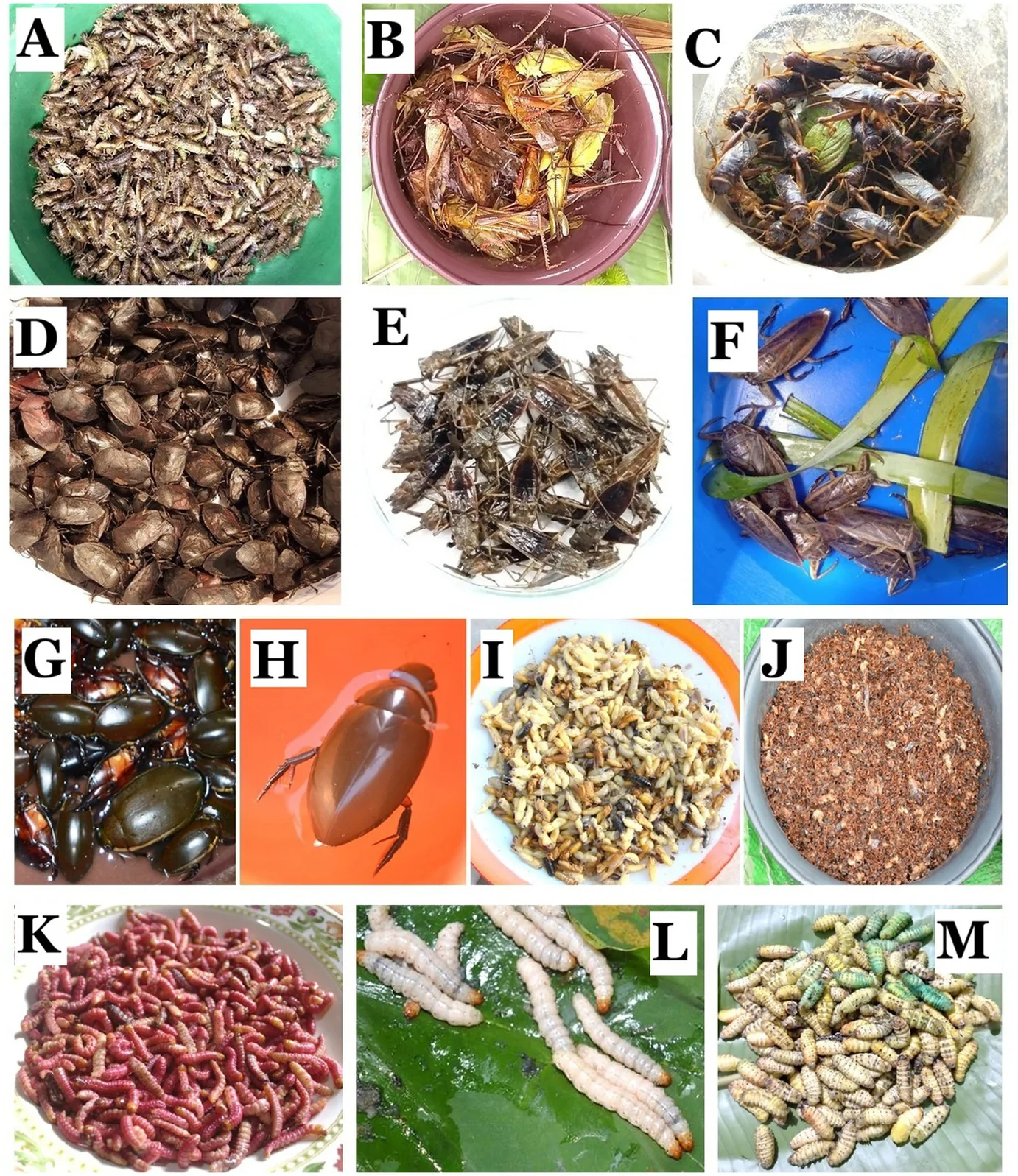
Edible insect species selected for nutritional analysis: **(A)** dragonfly nymphs; **(B)** *Mecopoda elongata*; **(C)** *Tarbinskiellus portentosus*; **(D)** *Coridius singhalanus*; **(E)** *Laccotrephes ruber*; **(F)** *Lethocerus indicus*; **(G)** *Cybister limbatus*; **(H)** *Hydrophilus caschmirensis*; **(I)** *Provespa barthelemyi* (all stages); **(J)** *Oecophylla smaragdina* (worker); **(K)** *Cossus* sp.; **(L)** *Omphisa fuscidentalis*; and **(M)** *Samia cynthia ricini*.

For amino-acid and fatty-acid profiling, three insect samples were pooled, homogenized, and processed as a single composite sample, which was subsequently subjected to HPLC and GC–MS analysis, respectively.

### 2.2 Proximate composition and biochemical analyses

The moisture and ash contents were determined according to the standard methods of the AOAC (26). The moisture content was measured by oven drying at 100 ± 5 °C to a constant weight, whereas the ash content was determined via incineration in a muffle furnace at 600 °C. The crude protein content was estimated via the Kjeldahl method (27) with a Kel Plus automatic digestion and distillation system (Pelican Equipment) and calculated via a nitrogen-to-protein conversion factor of 6.25. The total lipid content was determined via the sulpho-phospho-vanillin (SPV) method (28). The total carbohydrate content was estimated via the phenol–sulfuric acid (29) method. The total phenolic content was determined via the Folin–Ciocalteu method (30), and the total antioxidant content was assessed via the phosphomolybdenum assay (31). The absorbance for all colorimetric assays was measured via a Spectra Max Plus Microplate Reader and a Thermo Scientific Multiskan Go Microplate Spectrophotometer, and concentrations were calculated from the corresponding standard calibration curves.

The amino acid composition was determined according to Dhillon et al. (32).

For the analysis, 100 mg of insect sample was lyophilized for 90 min and hydrolyzed by vapor-phase hydrolysis with 200 μL of constant-boiling 6 N HCl and 40 μL phenol at 114 ± 2 °C for 24 h. The hydrolysates were dried, vacuum-dried for 90 min, and reconstituted in 200 μL of 20 mM HCl. A 20 μL aliquot was derivatized with 60 μL AccQ-Fluor borate buffer and 20 μL AccQ-Fluor reagent and heated at 55 °C. Amino acids were separated by HPLC (Waters 2,707 Module with PDA 2998), detected at 254 nm at 37 °C, and quantified using Empower Pro Software^®^ against Amino Acid Standard H; the results were expressed as μg 100 g^−1^. Fatty acid profiles were determined following Kumar and Dhillon (33). Briefly, 100 mg of insect sample was extracted with 10 mL chloroform:hexane (8:5:2, v/v/v), evaporated under nitrogen at 60 °C for 30 min, and converted to fatty acid methyl esters. The derivatives were analyzed by GC–MS (Shimadzu GCMS-QP2010 Ultra) using an Rtx^®^-5 MS column at a split ratio of 20:1, and the results were expressed as μg 100 g^−1^.

### 2.3 Data analysis

All experiments were performed in triplicate, and the results are presented as mean ± SD. Each insect species or species–developmental-stage combination was considered as a separate group. We evaluated differences among the 15 groups using one-way analysis of variance (ANOVA), followed by Tukey’s HSD test for multiple pairwise comparisons using GraphPad Prism 5.0. The larval, pupal, and adult stages of *Provespa bartheleymi* were treated as distinct groups and were not pooled. Statistical significance was considered at *p* < 0.05. Data normality was assessed using the Shapiro–Wilk test. As the data deviated from normality, Spearman’s rank correlation coefficient (ρ) was used to evaluate associations among the measured parameters. Principal component analysis (PCA) was performed separately for proximate/biochemical parameters, essential amino acids, non-essential amino acids, and fatty acids. For each insect species/developmental-stage group, the mean values obtained from three technical replicates (*n* = 3) were used as observations. PCA was performed based on the correlation matrix to account for differences in measurement units and scales among the variables. Spearman’s rank correlation, PCA, and graphical visualizations, including chord diagrams, were performed via OriginPro 2021 (Origin Lab Corporation, Northampton, MA, United States).

## 3 Results

The proximate compositions of the thirteen edible insect species are comprehensively summarized in Table 1.

**Table 1:** Proximate composition of thirteen commonly consumed edible insects.

Sl. No.	Order	Insect name	Stage	Moisture (%)	Ash (%)	Protein (%)	Lipid (%)	Carbohydrate (%)	Phenol (mg GAE g^−1^)	Antioxidant (mg AAE g^−1^)
1	Odonata	Dragonfly	Nymph	17.33 ± 0.06^a^	2.13 ± 0.42^a^	40.60 ± 0.59^ab^	12.06 ± 0.56^e^	0.79 ± 0.16^ef^	9.63 ± 0.36^bc^	15.02 ± 1.34^b^
2	Orthoptera	*Mecopoda elongata*	Adult	34.40 ± 0.43^bc^	1.52 ± 0.03^b^	18.90 ± 0.30^a^	4.31 ± 0.84^e^	1.74 ± 0.35^d^	12.30 ± 1.33^ab^	18.43 ± 3.68^b^
3		*Tarbinskiellus portentosus*	Adult	31.17 ± 0.99^bc^	1.47 ± 0.03^bc^	59.15 ± 1.15^abc^	25.13 ± 0.72^bc^	0.26 ± 0.05^f^	3.30 ± 0.12^f^	6.51 ± 0.36^c^
4	Hemiptera	*Coridius singhalanus*	Adult	34.37 ± 1.31^bc^	0.82 ± 0.10^d^	40.25 ± 1.37^ab^	35.08 ± 4.18^a^	0.14 ± 0.03^f^	6.32 ± 1.09^de^	4.01 ± 0.07^cd^
5		*Laccotrephes ruber*	Adult	42.43 ± 1.53^d^	2.07 ± 0.14^a^	63.91 ± 0.88^abc^	16.92 ± 2.28^c^	0.24 ± 0.02^f^	4.72 ± 2.33^de^	2.60 ± 0.21^d^
6		*Lethocerus indicus*	Adult	57.33 ± 0.36^f^	1.04 ± 0.15^cd^	56.90 ± 3.16^abc^	19.43 ± 2.12^bc^	0.14 ± 0.07^f^	4.11 ± 0.35^de^	2.01 ± 0.48^d^
7	Coleoptera	*Cybister limbatus*	Adult	35.92 ± 1.19^bc^	1.13 ± 0.11^bcd^	58.95 ± 1.34^abc^	16.12 ± 0.61^e^	0.26 ± 0.15^f^	2.80 ± 0.25^f^	3.13 ± 0.60^cd^
8		*Hydrophilus caschmirensis*	Adult	48.28 ± 0.42^e^	1.18 ± 0.16^bcd^	54.57 ± 0.51^abc^	20.01 ± 0.82^c^	0.32 ± 0.05^f^	8.59 ± 0.31^bc^	9.74 ± 1.26^c^
9	Hymenoptera	*Provespa bartheleymi*	Larva	33.57 ± 2.42^bc^	0.70 ± 0.20^d^	49.90 ± 1.75^ab^	25.79 ± 2.20^bc^	5.50 ± 0.90^b^	11.03 ± 0.96^ab^	17.45 ± 2.84^b^
			Pupa	28.76 ± 0.98^b^	0.87 ± 0.21^d^	48.15 ± 0.88^ab^	20.87 ± 5.68^bc^	3.54 ± 0.26^f^	7.73 ± 0.36^cd^	45.11 ± 3.25^a^
			Adult	29.26 ± 2.00^b^	0.95 ± 0.05^d^	68.28 ± 0.88^a^	19.93 ± 2.72^bc^	1.46 ± 0.07^e^	6.61 ± 0.13^cd^	19.43 ± 1.06^b^
10		*Oecophylla smaragdina*	Worker	20.43 ± 0.15^a^	0.81 ± 0.02^d^	53.99 ± 2.67^abc^	17.53 ± 2.68^bc^	7.72 ± 0.23^a^	13.53 ± 1.38^a^	7.72 ± 1.44^c^
11	Lepidoptera	*Cossus* sp.	Larva	54.77 ± 0.41^f^	0.79 ± 0.03^d^	36.18 ± 1.34^ab^	20.55 ± 0.44^bc^	0.28 ± 0.04^f^	3.90 ± 1.28^ef^	2.06 ± 0.38^d^
12		*Omphisa fuscidentalis*	Larva	38.97 ± 0.97^c^	0.88 ± 0.05^d^	30.35 ± 1.34^a^	17.89 ± 0.81^bc^	2.55 ± 0.34^c^	13.89 ± 0.93^a^	13.00 ± 1.00^bc^
13		*Samia cynthia ricini*	Larva	25.30 ± 0.38^ab^	1.05 ± 0.11^cd^	20.65 ± 0.98^a^	22.91 ± 5.92^bc^	0.41 ± 0.02^f^	9.53 ± 0.50^bc^	7.33 ± 0.44^c^

Values are expressed as Mean±SD (*n* = 3). Moisture and ash content were expressed on a fresh-weight basis, while all other proximate components were expressed on a dry-weight basis. Means with different superscript letters within columns differ significantly at *p* < 0.05 according to Tukey’s HSD test.

### 3.1 Macronutrient composition of edible insects

The moisture and ash contents varied considerably among the studied edible insects, reflecting species- and developmental stage-specific differences. The moisture content ranged from 17.33 ± 0.06% in dragonfly nymphs to 57.33 ± 0.36% in *L. indicus,* with high values also recorded in *Cossus* sp. larvae and *H. caschmirensis*. In contrast, the ash content ranged from 0.70 ± 0.20% in *P. bartheleymi* larvae to 2.13 ± 0.42% in dragonfly nymphs, followed by *L. ruber* (2.07 ± 0.14%). Developmental stage-specific variation was observed in *P. bartheleymi*, with larvae containing higher moisture and lower ash contents than pupae and adults, indicating physiological changes during metamorphosis. Lepidopteran larvae presented high moisture and moderate ash contents, which are consistent with previous reports of species- and habitat-dependent variation in edible insects, whereas Hemiptera presented the widest variation in moisture content.

The crude protein content varies markedly among insect species, reflecting species-specific nutritional differences. Within Orthoptera, protein content ranged from 18.90% in *M. elongata* to 59.15% in *T. portentosus*, whereas that of Hemiptera ranged from 40.25% in *C. singhalanus* to 63.91% in *L. ruber*. Coleoptera showed comparatively limited variation (54.57–58.95%). In Hymenoptera, the protein content increased from the larval to the adult stage, reaching a maximum of 68.28% in adult *P. bartheleymi*, suggesting developmental changes in protein accumulation. Lepidoptera exhibited relatively lower protein levels (20.65–36.18%), with *S. c. ricini having* the lowest value and *Cossus* sp. the highest within the order.

The lipid content varied substantially among species and developmental stages. In Orthoptera, the lipid content ranged from 4.31% in *M. elongata* to 25.13% in *T. portentosus*, whereas Hemiptera presented higher values (16.92–35.08%), with *C. singhalanus* recording the highest level. Coleoptera presented relatively limited variation (16.12–20.01%). In Hymenoptera, the lipid content was greater in larvae than in adults, peaking at 25.79% in larval *P. bartheleymi*. Lepidoptera presented the highest lipid levels, ranging from 22.91% in *S. c. ricini* to 64.29% in *Cossus* sp., highlighting the lipid-rich nature of the larval stages. The carbohydrate content was also low, ranging from 0.14–7.72%. The highest carbohydrate content was recorded in worker *O. smaragdina* (Table 1).

### 3.2 Total phenolic and antioxidant contents

The total phenolic content varied substantially among species and developmental stages, ranging from 2.80 mg GAE g^−1^ in *C. limbatus* to 13.89 mg GAE g^−1^ in *O. fuscidentalis*. Orthoptera (3.30–12.30 mg GAE g^−1^), Coleoptera (2.80–8.59 mg GAE g^−1^), and Lepidoptera (3.90–13.89 mg GAE g^−1^) presented considerable interspecific variation, whereas Hemiptera presented a narrower range (4.11–6.32 mg GAE g^−1^). In Hymenoptera, the phenolic content was highest in worker *O. smaragdina* (13.53 mg GAE g^−1^) and lowest in adult *P. bartheleymi* (6.61 mg GAE g^−1^), indicating developmental variation. The antioxidant content varied markedly among species and developmental stages, ranging from 2.01 mg AAE g^−1^ in *L. indicus* to 45.11 mg AAE g^−1^ in pupal *P. bartheleymi*. Orthoptera (6.51–18.43 mg AAE g^−1^) and Coleoptera (3.13–9.74 mg AAE g^−1^) presented moderate variation, whereas Hemiptera consistently presented low antioxidant capacity (2.01–4.01 mg AAE g^−1^). In Hymenoptera, the antioxidant capacity was highest in the pupal stage of *P. bartheleymi* and lowest in the worker *O. smaragdina* (7.72 mg AAE g^−1^), indicating developmental variation. Lepidoptera presented values ranging from 2.06 to 13.00 mg AAE g^−1^.

### 3.3 Integrated analysis of nutritional and bioactive properties

Considerable variation in nutritional and bioactive potential was observed among the investigated samples, indicating distinct species-specific compositional profiles (Figure 3). The differential connections between individual species and protein, lipid, carbohydrate, phenolic, and antioxidant content highlighted the heterogeneous nature of their nutritional and functional potential. These compositional patterns were further explored using Spearman’s rank correlation analysis (Figure 4). Strong positive correlations were observed between carbohydrate and phenolic content (ρ = 0.782) and between carbohydrate and antioxidant content (ρ = 0.784). Phenolic content was also positively correlated with antioxidant content (ρ = 0.629). Conversely, moisture showed moderate negative correlations with carbohydrate (ρ = −0.513) and antioxidant content (ρ = −0.568). Ash content was negatively associated with lipid content (ρ = −0.557), whereas protein exhibited a moderate negative correlation with phenolic content (ρ = −0.532). The remaining relationships were weak or negligible.

**Figure 3:**
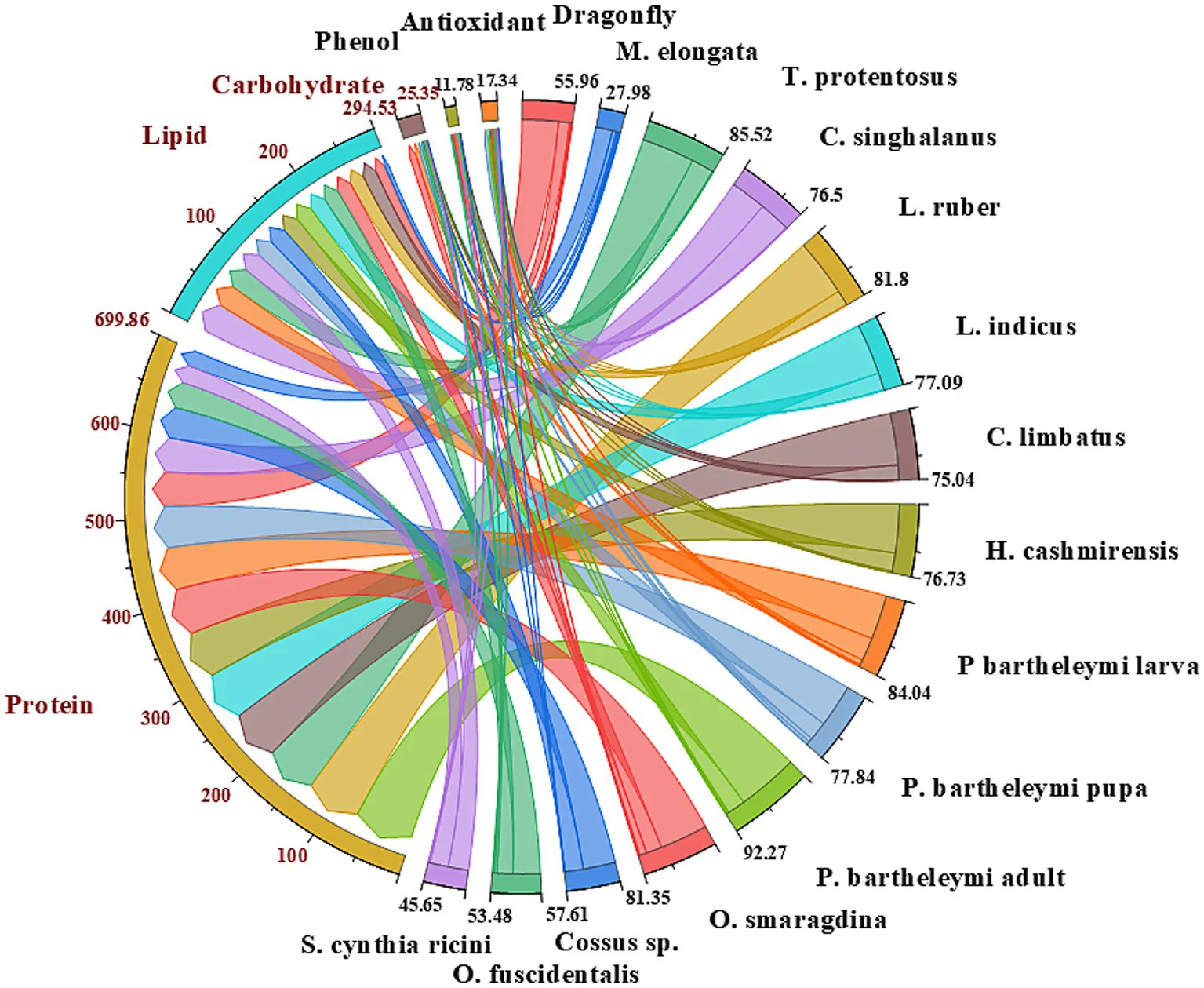
Chord diagram illustrating the relationships between 15 insect groups and their nutritional and bioactive characteristics, including protein, lipid, carbohydrate, phenolic, and antioxidant content. The width of each chord represents the relative contribution of individual species to the corresponding nutritional parameter, whereas the sector size reflects the overall magnitude of each attribute across the studied species. The diagram highlights species-specific variation in nutrient composition and bioactive properties.

**Figure 4:**
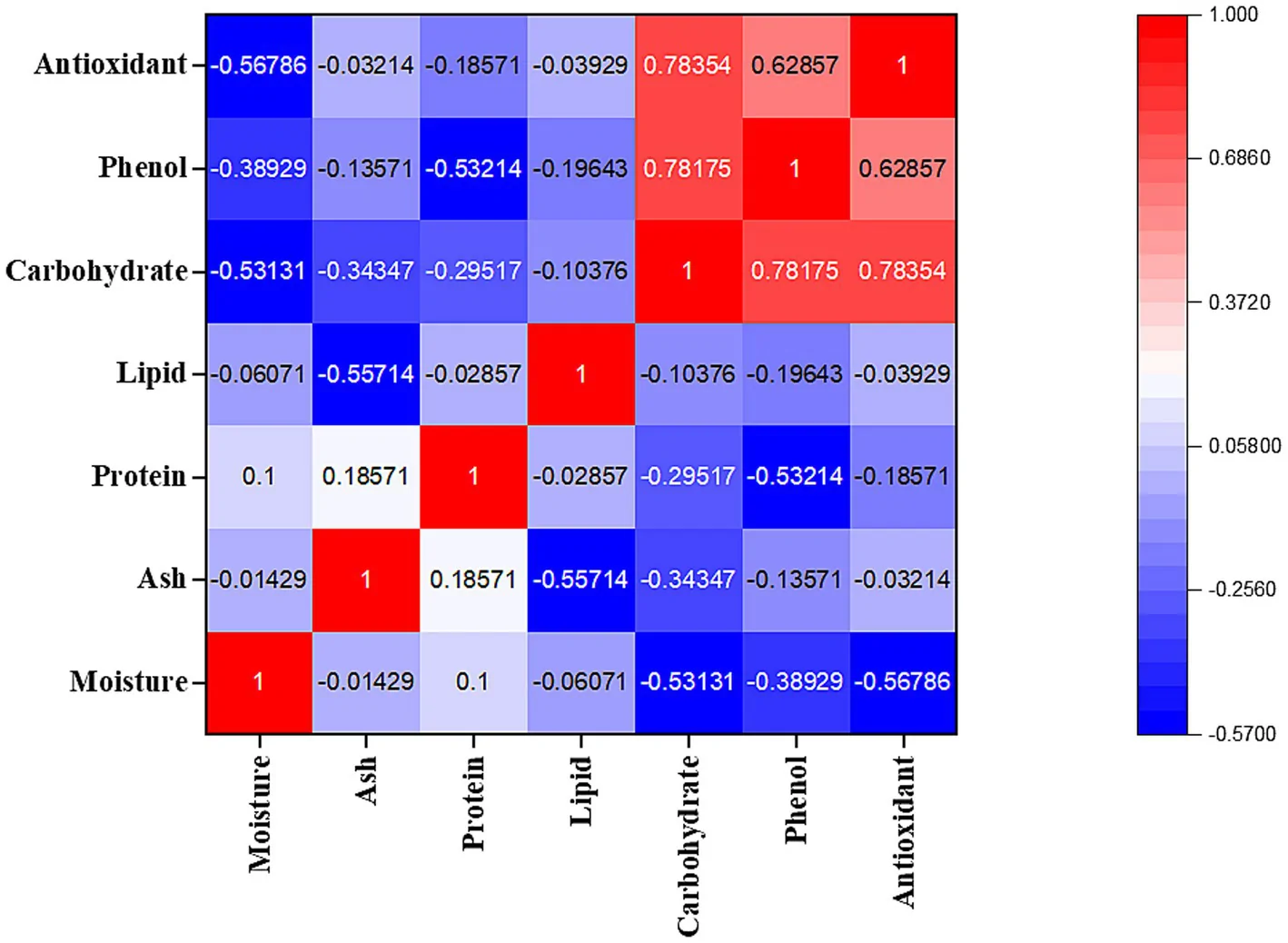
Spearman’s correlation heatmap showing relationships among proximate composition, phenolic and antioxidant content.

### 3.4 Amino acid profile

Eight essential amino acids (EAAs) and nine nonessential amino acids (NEAAs) were detected across all edible insect species, with substantial interspecific and developmental variation (Table 2). Leucine was the predominant EAA in most species, whereas valine and threonine predominated in selected taxa. Histidine was consistently the least abundant EAA. Larval *P. bartheleymi* presented the highest concentrations of several EAAs, including isoleucine, leucine, lysine, methionine, phenylalanine, and valine, indicating a nutritionally rich amino acid profile. Among the NEAAs, proline predominated in most species, whereas alanine was highest in *C. singhalanus*, *C. limbatus*, pupal *P. bartheleymi*, and *O. smaragdina*. Larval stages, particularly *Cossus* sp. and *P. bartheleymi*, presented marked enrichment of several NEAAs, highlighting pronounced species- and stage-specific variation in amino acid composition.

**Table 2:** Amino acid profile per 100 g of insect sample.

Insect	Stage	Essential amino acids (EAA) in μg 100 g^−1^ of the insect sample								Non-essential amino acids (NEAA) in μg 100 g^−1^ of the insect sample								
		His	Ile	Leu	Lys	Met	Phe	Thr	Val	Ala	Asp	Arg	Cys	Glu	Gly	Pro	Ser	Tyr
Dragonfly	Nymph	4.08	21.34	26.82	12.21	10.58	12.92	12.63	35.69	31.08	2.37	17.25	5.91	ND	2.73	34.56	1.62	19.06
*M. elongata*	Adult	0.47	4.17	4.42	1.53	1.51	1.15	1.36	5.08	5.77	2.15	3.13	1.65	1.22	3.43	9.84	0.56	2.16
*T. portentosus*	Adult	7.48	25.74	48.06	20.00	20.20	36.75	23.71	29.56	39.20	14.40	32.84	7.90	ND	35.72	42.57	2.94	11.25
*C. singhalanus*	Adult	0.38	8.89	13.56	4.19	4.41	6.23	21.78	14.59	29.49	18.05	13.47	2.49	ND	18.76	25.49	4.33	9.84
*L. ruber*	Adult	0.54	12.56	38.29	6.87	7.36	10.18	0.85	18.81	10.17	17.30	3.29	5.44	ND	7.12	18.01	0.93	5.72
*L. indicus*	Adult	1.69	3.02	4.55	2.90	4.70	2.37	5.13	4.26	7.15	2.52	3.90	4.61	0.76	9.03	12.19	1.87	3.98
*C. limbatus*	Adult	1.59	5.44	6.06	7.32	3.78	4.13	5.59	7.83	16.00	3.51	17.25	ND	ND	2.73	15.79	1.02	1.37
*H. caschmirensis*	Adult	1.88	11.07	16.09	5.30	4.00	7.22	7.28	12.88	20.00	4.11	3.94	1.80	ND	9.20	28.59	1.25	1.95
*P. bartheleymi*	Larva	22.57	78.42	81.09	46.79	47.59	97.00	27.89	61.18	55.57	12.07	37.93	26.36	8.29	41.02	92.35	3.63	34.40
	Pupa	1.94	16.65	28.92	18.58	5.46	19.41	3.97	12.73	22.66	9.65	10.13	2.69	0.24	22.63	20.30	1.17	8.51
	Adult	4.57	19.11	24.90	5.25	11.34	18.26	9.13	23.35	19.83	8.03	17.41	5.06	ND	33.76	19.98	0.85	35.39
*O. smaragdina*	Worker	9.10	45.78	47.51	14.03	21.48	43.32	12.81	33.23	31.85	9.41	24.95	24.01	1.17	25.67	24.27	1.79	27.82
*Cossus* sp.	Larva	25.75	53.25	78.37	21.58	28.44	57.43	35.35	52.4	88.23	7.14	44.37	11.47	5.83	112.08	76.09	4.64	64.67
*O. fuscidentalis*	Larva	1.06	2.37	3.00	2.68	1.55	1.20	13.09	4.71	7.39	1.04	4.77	ND	0.47	4.41	7.51	1.89	2.60
*S. cynthia ricini*	Larva	1.03	1.21	1.99	1.96	2.31	1.11	3.53	2.42	7.60	2.29	3.13	2.52	0.31	9.47	10.56	2.26	3.04

EAA: His, Histidine; Ile, Isoleucine; Leu, Leucine; Lys, Lysine; Met, Methionine; Phe, Phenylalanine; Thr, Threonine; Val, Valine; NEAA: Ala, Alanine; Asp, Aspartic acid; Arg, Arginine; Cys, Cysteine; Glu, Glutamic acid; Gly, Glycine; Pro, Proline; Ser, Serine; Tyr, Tyrosine; ND, Not Detected.

### 3.5 Fatty acid profile

Six major fatty acids were identified across the studied insects, comprising three saturated (myristic, palmitic, and stearic), two monounsaturated (palmitoleic and oleic), and one polyunsaturated (linoleic) fatty acid (Table 3). The total saturated fatty acid content ranged from 29.69 to 42.50 μg 100 g^−1^, the monounsaturated fatty acid content ranged from 46.28 to 56.90 μg 100 g^−1^, and the polyunsaturated fatty acids from 0.11 to 20.12 μg 100 g^−1^. Oleic acid was the predominant monounsaturated fatty acid, reaching its highest concentration in *Cossus* sp., whereas linoleic acid was most abundant in dragonfly nymphs. Palmitic acid was highest in *O. fuscidentalis*, myristic acid in *H. caschmirensis*, and stearic acid in pupal and adult *P. bartheleymi*.

**Table 3:** Fatty acid profiles of the thirteen edible insect species.

Insect	Stage	Fatty acid (μg 100 g^−1^)								
		Myristic acid	Palmitic acid	Stearic acid	Palmitoleic acid	Oleic acid	Linoleic acid	TSFA	TMUFA	TPUFA
Dragonfly	Nymph	1.41	21.33	6.95	4.64	41.64	20.12	29.69	46.28	20.12
*M. elongata*	Adult	0.54	20.26	11.16	1.93	52.44	8.57	31.96	54.37	8.57
*T. portentosus*	Adult	0.78	26.00	5.22	7.54	42.11	18.36	32.00	49.65	18.36
*C. singhalanus*	Adult	0.83	32.11	5.83	13.64	42.52	4.71	38.77	56.16	4.71
*L. ruber*	Adult	0.75	33.19	7.87	8.18	41.08	8.55	41.81	49.26	8.55
*L. indicus*	Adult	0.53	21.17	11.69	1.80	53.36	8.21	33.39	55.16	8.21
*C. limbatus*	Adult	1.15	31.64	7.27	4.47	42.55	10.99	40.06	47.02	10.99
*H. caschmirensis*	Adult	2.86	30.34	6.07	11.62	38.74	7.41	39.27	50.36	7.41
*P. bartheleymi*	Larva	2.30	28.72	9.06	1.88	51.89	5.05	40.08	53.77	5.05
	Pupa	2.77	25.69	9.73	1.75	53.02	7.04	38.19	54.77	7.04
	Adult	1.88	24.41	12.39	0.67	50.56	8.10	38.68	51.23	8.10
*O. smaragdina*	Worker	2.21	33.14	7.15	3.82	51.42	1.82	42.50	55.24	1.82
*Cossus* sp.	Larva	0.67	28.00	5.27	0.86	56.04	0.11	33.94	56.90	0.11
*O. fuscidentalis*	Larva	0.76	38.77	1.45	13.20	39.22	4.56	40.98	52.42	4.56
*S. cynthia ricini*	Larva	1.94	27.99	6.98	9.80	36.68	7.17	36.91	46.48	7.17

TSFA, Total Saturated Fatty Acid; TMUFA, Total Monounsaturated Fatty Acid; TPUFA, Total Polyunsaturated Fatty Acid.

### 3.6 Multivariate analysis of nutritional and functional characteristics

PCA revealed clear differences in the nutritional profiles of the studied insects, with PC1 and PC2 explaining 36.16 and 23.36% of the total variation. Species such as *C. singhalanus*, *L. indicus,* and *T. protentosus* were associated with lipid, protein, and moisture, whereas *O. smaragdina*, *P. barthelemyi,* and *O. fuscidentalis* were more closely associated with carbohydrate, phenol, and antioxidant contents. Species *L. ruber*, dragonfly, and *M. elongata* were separated along PC2, indicating distinct nutritional profiles (Figure 5A). The PCA of essential amino acids explained 96.67% of the total variation and clearly separated *P. barthelemyi* larvae from the other species because of their strong association with lysine, leucine, isoleucine, methionine, phenylalanine, histidine, and valine (Figure 5B). Similar results were obtained for nonessential amino acids, with the PCA explaining 77.71% of the total variance, and *P. barthelemyi* larvae were highly associated with glycine, proline, alanine, glutamic acid, cysteine, arginine, and tyrosine, whereas *C. singhalanus* was more associated with aspartic acid and serine (Figure 5C).

**Figure 5:**
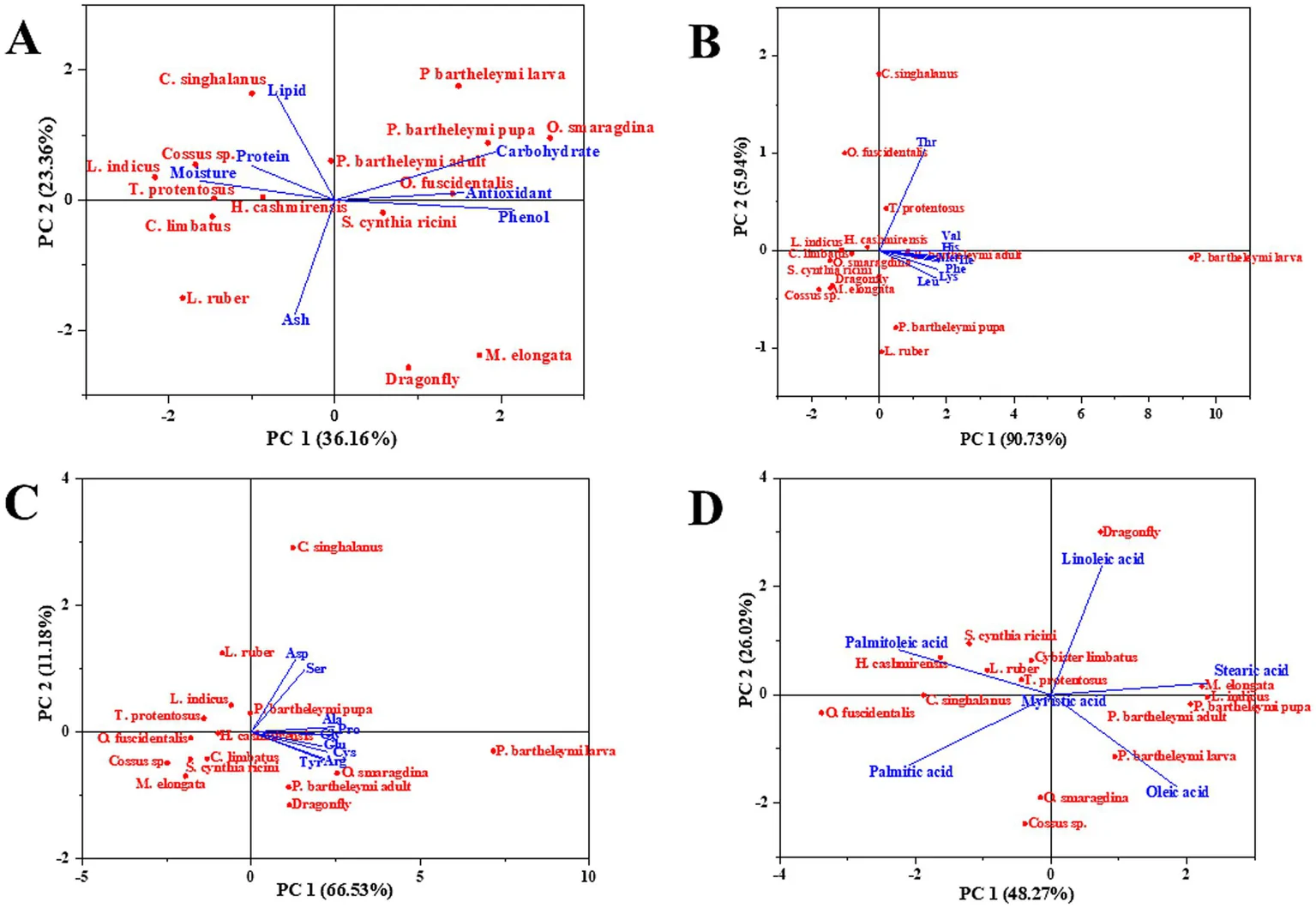
Multivariate analysis of the nutritional and bioactive characteristics of 13 edible insect species. **(A)** Principal component analysis (PCA) biplot of nutritional and bioactive parameters. **(B)** Essential amino acid composition. **(C)** Nonessential amino acid composition. **(D)** Fatty acid composition. The PCA plots illustrate the distribution of insect species based on their compositional profiles and the contributions of individual nutritional variables to species differentiation.

The PCA of fatty acid composition explained 74.29% of the total variation, with PC1 and PC2 accounting for 48.27 and 26.02% of the variance, respectively (Figure 5D). *P. barthelemyi* larvae were strongly associated with oleic acid, whereas dragonfly nymphs were closely associated with linoleic acid. On the other hand, stearic acid was associated with *M. elongata*, *L. indicus,* and *P. barthelemyi* (adult and pupa), and palmitoleic acid was associated with *H. cashmirensis*, *S. c. ricini*, and *C. limbatus*. Multivariate analyses generally revealed interspecific differences in nutritional composition and bioactivity. Across the species studied, the larvae of *P. barthelemyi* were consistently positively associated with essential amino acids, nonessential amino acids, and unsaturated fatty acids, suggesting their nutritional importance. *O. smaragdina* and *O. fuscidentalis* were characterized by their associations with phenolic content and antioxidant content, indicating their potential functional food value.

## 4 Discussion

### 4.1 Edible insects: protein- and lipid-rich, low-carbohydrate resources

Proteins play a fundamental role in growth, tissue repair, and immune function, and the present study revealed substantial variation in crude protein content among edible insect taxa and developmental stages, which is consistent with previous reports (34). This variability occurred both within and between taxonomic groups, reflecting the influence of species-specific physiology, developmental stage, and environmental conditions on nutrient composition. For example, *T. portentosus* (59.15%) contained considerably more protein than did *Velarifictorus asperses* (46.31%) and *Gryllotalpa africana* (12.9%), corroborating pronounced interspecific variation within Orthoptera (35, 36). Similarly, adults of *Zonocerus variegatus* contain more protein than immature stages do, whereas early instars are richer in minerals, highlighting the influence of development on nutritional quality (37). Although the present study focused on wild-harvested insects, differences in natural diet and habitat conditions may have further contributed to the observed variation. Nutritional quality can also be improved through dietary manipulation; sunflower seed cake-based diets increase protein, mineral, and linoleic acid contents in *Ruspolia differens*, demonstrating the potential of agro-industrial byproducts to produce nutritionally enriched insects while supporting circular food systems (38). The protein contents observed in the present study fall within the reported ranges for major edible insect orders and are comparable to, or exceed, those of many conventional protein sources. Coupled with their generally balanced essential amino acid composition, these findings reinforce the potential of edible insects as sustainable, high-quality protein sources for future food systems (22, 39).

In the major nutritional fraction after protein, greater lipid accumulation with considerable variation across taxa and developmental stages reflects their role in energy storage during growth and metamorphosis (40). Consistent with earlier reports (22, 34), the present findings demonstrate that species such as *T. portentosus* and *C. singhalanus* contain relatively higher lipid levels than related taxa do (35, 41, 42), thereby reinforcing their potential as energy-dense nutritional resources. Within the global discourse on sustainable protein sources, the present findings from Nagaland are particularly significant, as they integrate region-specific evidence with broader nutritional perspectives. The state’s rich entomofaunal diversity, together with deeply rooted entomophagy practices among indigenous communities, represents a valuable yet underexplored reservoir of nutritionally important bioresources.

Although the carbohydrate content remained consistently low across all the examined taxa, it clearly presented interspecific variation. Among orthopterans, *M. elongata* (1.74%) presented a comparatively higher carbohydrate content than several related species, such as *Eupreponotus inflatus* (0.27%), *C. robustus* (0.29%), *C. rosea* (0.21%), and *O. fuscovittata* (0.37%) reported in earlier studies by Narzari (43), suggesting possible species-specific metabolic or dietary influences. In contrast, *C. singhalanus* (0.17%) and *C. limbatus* (0.26%) presented markedly lower carbohydrate levels, falling within or below the lower range documented for comparable orthopteran taxa (43–45). A similar pattern was observed among hymenopterans; *P. bartheleymi* presented a relatively high carbohydrate range (1.46–5.5%), consistent with previous findings reporting moderate variability in carbohydrate reserves within this group (43). The observed interspecific variation in carbohydrate content across taxa may be influenced by species-specific and developmental factors; however, ecological drivers such as diet were not directly evaluated in this study.

Despite clear interspecific variation, the carbohydrate fraction across the studied species remained consistently low relative to other proximate components, particularly proteins and lipids. This pattern highlights a fundamental nutritional characteristic of edible insects: they do not constitute a substantial source of dietary carbohydrates. Instead, their nutritional value is derived primarily from their high protein and lipid contents, emphasizing their role as nutrient-dense foods rather than carbohydrate-based energy sources. The protein levels documented in the present study are in close agreement with global reports (46–48), further reinforcing the recognition of edible insects as high-quality protein sources. The present findings underscore the practical importance of insects as accessible, culturally acceptable, and nutritionally reliable food resources, particularly in addressing food and nutritional security in resource-limited settings. By bridging local knowledge systems with scientific validation, this study strengthens ongoing efforts to develop resilient, nutritionally adequate, and ecologically sustainable food systems.

### 4.2 Edible insects for functional food innovation

The compositional profile of edible insects has important implications for food product development, particularly within the framework of value addition and functional food design. In general, edible insects are characterized by low carbohydrate content alongside moderate to high levels of lipids and proteins, a combination that enhances their suitability as ingredients in nutrient-dense formulations (13, 22). The relatively low carbohydrate fraction, when coupled with substantial lipid reserves, contributes to increased caloric density, making insects especially valuable in the development of high-protein, high-energy food products intended for populations with increased nutritional demands (49–52). The lipid fraction of insects plays a critical role not only in energy provision but also in determining the physicochemical and sensory properties of food products. Insect-derived lipids can improve texture, enhance palatability, and contribute to desirable flavor profiles and mouthfeel, thereby increasing consumer acceptability (53, 54). These functional attributes are particularly advantageous in the formulation of value-added products such as fortified flours, protein-enriched bakery goods, snack items, ready-to-eat foods, and nutraceutical preparations, where both nutritional quality and sensory appeal are essential for market success. Furthermore, the marked variability in lipid content across insect species and developmental stages offers considerable scope for targeted application in food formulations. Larval stages, which typically exhibit higher fat content, can be strategically utilized in energy-dense products, whereas adult forms with comparatively lower lipid levels are more appropriate for protein-centric formulations requiring controlled fat content (23). Such stage-specific and species-specific variability enables the customization of ingredient profiles to meet specific dietary or functional requirements. Importantly, the inherently low carbohydrate content of insects provides flexibility in formulation strategies. Insects can be effectively blended with carbohydrate-rich raw materials, such as cereals and tubers, to develop nutritionally balanced food products with optimized macronutrient compositions. This integrative approach not only enhances the nutritional profile but also aligns with current trends in the development of sustainable, high-quality alternative protein sources for food security and functional nutrition.

The protein quality of edible insects remains a central determinant of their nutritional value. The present findings are consistent with previous reports indicating that insects provide high-quality protein with a relatively balanced amino acid profile comparable to that of conventional animal sources (55). However, certain essential amino acids, particularly lysine or tryptophan, may be limiting in some species (56), highlighting the importance of dietary complementation when insects are used as primary protein sources. Within this context, the present findings demonstrate that the nutritional profile of edible insects is highly dynamic and influenced by species-specific genetics, feeding substrates, ecological conditions, and developmental stages. Larval forms typically exhibit relatively high protein and amino acid concentrations due to active growth and nutrient accumulation, whereas adult stages reflect metabolic shifts associated with reproduction and maintenance (39, 53). Protein quality and amino acid availability are significantly influenced by postharvest processing and culinary practices. Processing methods can alter amino acid integrity and bioavailability, necessitating the optimization of techniques to preserve nutritional quality while enhancing functional attributes.

### 4.3 Edible insects as bioactive therapeutic sources

In addition to their conventional role as nutrient-rich food sources, edible insects are increasingly recognized as reservoirs of bioactive compounds with potential functional relevance, which requires further validation through bioavailability and clinical studies (50, 57, 58). Although phenolic compounds are well documented in plant-derived foods and products such as honey, where they are closely linked to antioxidant capacity and therapeutic efficacy (59, 60), their presence in edible insects has only recently gained systematic scientific attention. Current evidence indicates that insects can assimilate, sequester, and biotransform plant-derived secondary metabolites, thereby functioning as reservoirs of bioactive compounds (61–63). This highlights the importance of the ecological context, particularly diet and habitat, in determining the biochemical composition of insect taxa.

The present study provides region-specific insights into the phenolic content of edible insects, thereby contributing to a relatively underexplored area of insect-based nutritional research, particularly in the context of Northeast India. For example, the relatively high phenolic content observed in *L. ruber* relative to *L. maculatus*, and in *C. limbatus* and *H. caschmirensis* relative to previously reported congeners (45), indicates that phenolic accumulation is not only species-specific but also potentially influenced by habitat, diet, and the ecological niche. Similarly, the relatively lower TPC recorded in *Cossus* sp. compared to values reported by Aochen et al. (64) further highlights the sensitivity of phenolic quantification to extraction conditions, solvent polarity, and analytical techniques. In addition to intrinsic biological factors, postharvest processing has emerged as a critical determinant of phenolic retention and functional quality. Empirical evidence from studies on edible stink bugs (*Encosternum delegorguei*) has revealed significantly higher concentrations of total phenolics, flavonoids, and tannins in unprocessed samples than in their thermally processed counterparts, suggesting that conventional cooking methods may induce degradation or leaching losses of these compounds (65).

From a mechanistic perspective, the occurrence of phenolic compounds in insects is largely governed by dietary intake and subsequent metabolic transformation. Comparative studies have demonstrated significant overlap between host plant and insect phenolic profiles, supporting the concept of trophic transfer and biochemical integration (66). This relationship is particularly evident in phytophagous taxa, such as Lepidoptera, where dietary phenolics are incorporated into body tissues and contribute to antioxidant capacity. Such insights have important implications for insect farming and semi-domestication strategies, as controlled dietary interventions could be used to modulate phenolic content and enhance functional properties. Studies on edible ground crickets (*Henicus whellani*) have demonstrated measurable levels of phenolics, flavonoids, and tannins, supporting the hypothesis that the phenolic composition of insects is strongly shaped by their feeding ecology and trophic interactions (67).

Phenolic compounds have been associated with antioxidant activity and may contribute to the antioxidant capacity of foods through mechanisms such as free-radical scavenging and metal chelation (59, 67, 68). Insects may also contain other bioactive constituents, including alkaloids, terpenoids, and amino acid derivatives, which could contribute to their overall antioxidant properties. Antioxidant activity has previously been reported in insects belonging to orders such as Orthoptera, Coleoptera, and Lepidoptera, with phenolic compounds suggested as one of the contributing components (69). In the present study, phenolic content ranged from 2.80 ± 0.25 to 13.89 ± 0.93 mg GAE g^−1^, while antioxidant content varied from 2.01 ± 0.48 to 45.11 ± 3.25 mg AAE g^−1^. The highest antioxidant content was observed in *Provespa bartheleymi* pupa (45.11 ± 3.25 mg AAE g^−1^), despite its comparatively moderate phenolic content (7.73 ± 0.36 mg GAE g^−1^). Conversely, *Omphisa fuscidentalis* exhibited the highest phenolic content (13.89 ± 0.93 mg GAE g^−1^), but its antioxidant content was considerably lower (13.00 ± 1.00 mg AAE g^−1^). These findings indicate that phenolic compounds may contribute to the antioxidant capacity of edible insects, although the variation in antioxidant content suggests that other constituents may also be involved. Thus, the observed phenolic and antioxidant profiles support the potential functional value of these insects but do not, by themselves, establish therapeutic effects or specific health benefits. Further studies involving compound-specific characterization, bioavailability, and biological activity assays are needed to determine the functional relevance of these constituents.

### 4.4 Postharvest processing: key determinants of phenolic retention and functional quality

Ethnic communities of Nagaland, utilize a diverse range of traditional preparation methods for edible insects, including boiling in minimal water, frying in hot oil, cooking with locally available ingredients, roasting over open fire or hot charcoal, steaming, consuming them raw to retain their natural flavor, and processing them into chutney (Figure 6). Cooking with local ingredients is the preferred method, as it allows the integration of insects into familiar dishes; improves taste, aroma, and acceptability and ensures microbial safety through adequate heat treatment. Frying is widely favored because of its ability to enhance texture—imparting crispiness—and intensify flavor through lipid-mediated reactions, making insects more appealing, especially to younger consumers. Roasting over fire or charcoal is another popular method, as it imparts a smoky flavor. Boiling, although less preferred, is considered a simple and hygienic method that softens insects and reduces potential toxins or contaminants, albeit sometimes at the cost of flavor dilution. The consumption of raw insects is practiced in specific contexts where freshness is assured, allowing individuals to experience natural taste and texture, although this method may carry greater food safety risks. Preparation as a chutney represents a value-added method in which insects are ground with spices and condiments, improving flavor diversity and enabling incorporation into regular meals. Steaming is the least preferred method, possibly because of its relatively bland taste profile and limited flavor enhancement compared with other techniques. The observed preference hierarchy—cooked > fried > roasted > boiled > raw > chutney > steamed (70)—can thus be justified by a combination of sensory appeal, convenience, cultural familiarity, and food safety considerations. These varied culinary practices are deeply rooted in indigenous knowledge systems and are shaped by factors such as resource availability, cultural preferences, seasonal abundance, and the need to increase palatability, safety, and nutritional value.

**Figure 6:**
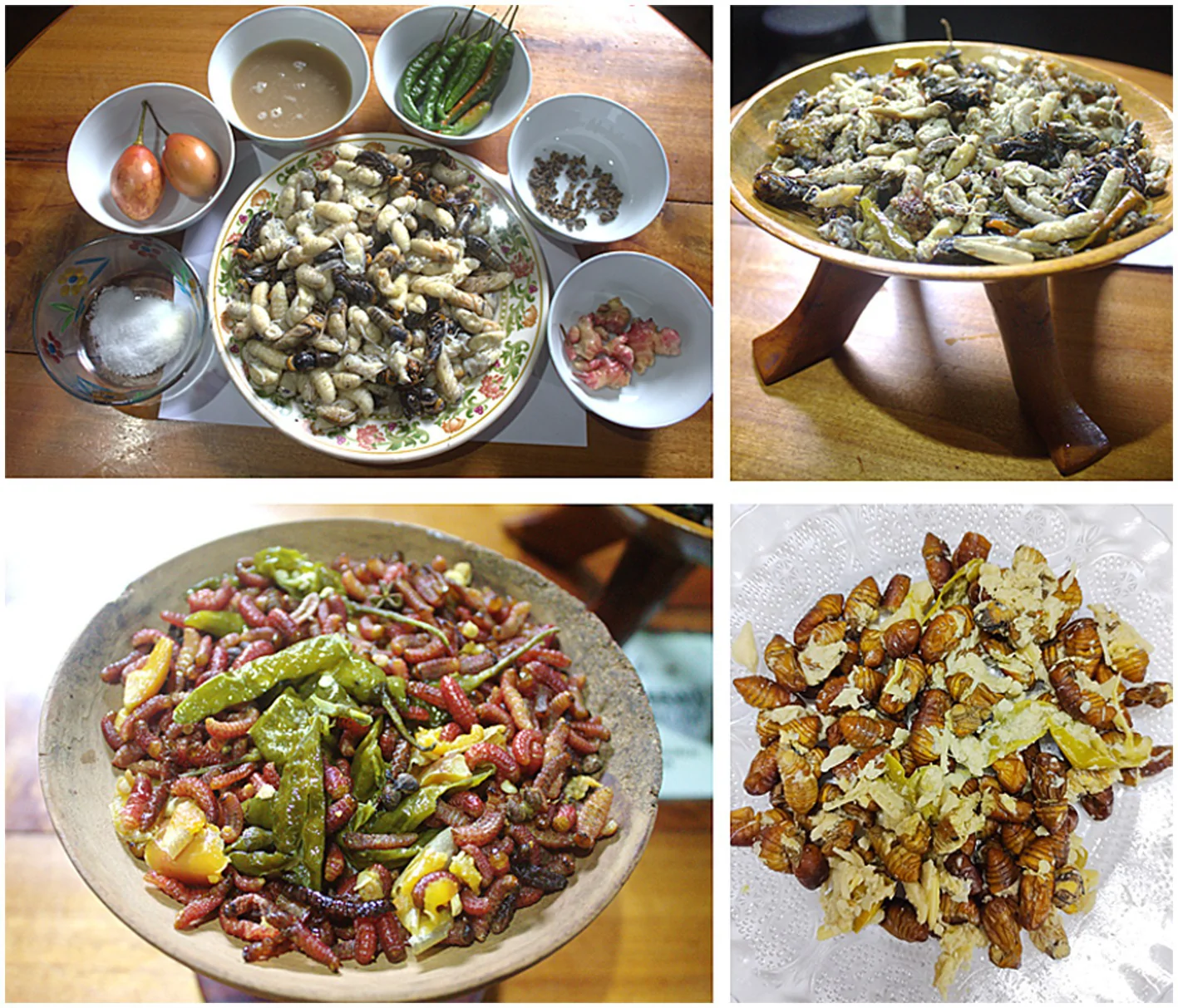
Traditional preparation methods of edible insects consumed in Nagaland, India. Top left: *Vespa mandarinia* (larvae, pupae, and adults) with local ingredients before cooking; Top right: cooked *V. mandarinia*; Bottom left: *Cossus* sp. prepared with local ingredients; Bottom right: eri silkworm (*Samia cynthia ricini*) pupae cooked with local ingredients.

Postharvest processing has emerged as a critical determinant of phenolic retention and functional quality. Given that edible insects are commonly subjected to processing techniques such as roasting, boiling, drying and extrusion, understanding the stability of phenolic compounds under these conditions is essential. While studies in plant systems suggest that optimized thermal treatments can preserve or even increase phenolic bioavailability under certain conditions (71–73), comparable systematic investigations in insect matrices remain limited, representing a significant research gap. Processing also exerts a dual influence on protein quality and functionality. Thermal treatments can induce protein denaturation and Maillard reactions, potentially reducing the bioavailability of essential amino acids such as lysine, while simultaneously increasing digestibility by disrupting chitin–protein complexes (40). These findings underscore the importance of optimizing processing conditions to balance nutrient preservation with improved bioaccessibility and sensory quality. More broadly, processing methods—including roasting, boiling, drying and frying—affect physicochemical properties such as protein structure, lipid stability and chitin interactions, thereby influencing nutrient availability, flavor development and consumer acceptance (24).

### 4.5 Intrinsic–extrinsic determinants of edible insect nutritional profile

The nutritional and bioactive composition of edible insects is influenced by a complex interaction of intrinsic biological factors and extrinsic environmental and technological conditions (Supplementary Figure 1). Species-specific characteristics, developmental stage, diet, habitat, and processing conditions can contribute to substantial variation in protein, lipid, carbohydrate, mineral, amino acid, fatty acid, phenolic, and other bioactive components. Meyer-Rochow et al. (24) emphasized that these factors can substantially influence nutrient allocation and composition, indicating that the nutritional characteristics of insects should not be regarded as fixed species-specific traits. The interspecific differences observed in the present study may therefore reflect the combined effects of species-specific biology and environmental conditions. Comparisons among studies are nevertheless challenging because of differences in reporting units, sample preparation, extraction procedures, and analytical methods, highlighting the need for standardized approaches.

Developmental stage is an important determinant of insect nutritional composition because physiological and metabolic requirements change throughout the life cycle. Duarte et al. (74) reported stage-dependent differences in *Tribolium castaneum*, with adults showing relatively higher protein, fiber, and nitrogen-free extract contents, whereas larvae contained higher concentrations of several minerals, including phosphorus, potassium, sulfur, and zinc. Changes in amino acid and fatty acid profiles across developmental stages further reflected differences in nutritional requirements associated with growth, metamorphosis, and adult activity. Similar developmental variation has been reported in *Blaberus craniifer* and *Zophobas morio*, although the magnitude and direction of these changes differed among species (75). These reports indicate that developmental effects are species-dependent and may influence both the quantity and quality of nutrients, including amino acid and fatty acid composition. Recent evidence further supports the influence of developmental transitions on nutritional and bioactive characteristics. Vehar et al. (76) observed substantial variation in amino acid, fatty acid, mineral, and polyphenol profiles among farmed insects, with developmental transitions contributing to changes in nutrient composition. Similarly, Liu et al. (77) reported pronounced stage-dependent changes in protein, lipid, fatty acid, vitamin, and mineral composition throughout the life cycle of *Hermetia illucens*, with notable nutrient accumulation during the mature larval and early prepupal stages. Toviho and Bársony (78) further demonstrated that developmental status and body size may be more informative than chronological age alone, as larger *Tenebrio molitor* larvae differed in dry matter, fiber, chitin, and nitrogen-free extract contents, while the transition to the pupal stage was accompanied by changes in dry matter, lipid, and chitin.

In addition to developmental stage, species-specific characteristics contribute substantially to nutritional variability. Tanga et al. (79) reported marked differences in proximate, fatty acid, mineral, and vitamin composition among *Gonimbrasia cocaulti*, *Bombyx mori*, and *Samia cynthia ricini*, despite the high protein content observed across the investigated insects. Such differences demonstrate that insects cannot be considered nutritionally equivalent solely on the basis of their high protein content, as their lipid, fiber, mineral, and fatty acid profiles may differ considerably. Diet and rearing conditions represent another major source of variation. Riekkinen et al. (80) demonstrated that the composition of rearing substrates can modify the protein, lipid, mineral, and particularly fatty acid profiles of edible insects. The availability of dietary fatty acids, including linoleic and α-linolenic acids, may influence their incorporation into insect biomass, although responses vary among species because of differences in nutrient assimilation and metabolism. Host plant–insect interactions are therefore particularly relevant, as dietary substrates can influence growth, biomass production, and biochemical composition, including phenolic and amino acid profiles. Controlled manipulation of host plants and rearing substrates may consequently provide opportunities for targeted improvement of nutritional quality and more consistent production.

The nutritional quality of edible insects is therefore determined by the combined effects of species identity, developmental physiology, diet, environmental conditions, and processing. These factors should be considered when interpreting interspecific differences and comparing results across studies. In particular, the identification of suitable host plants and optimization of controlled rearing systems may facilitate the production of insect biomass with desirable nutritional and functional characteristics. At the same time, integrating traditional knowledge with semi-domestication and improved harvesting practices could enhance productivity while reducing ecological disturbance and supporting biodiversity conservation (81, 82). A clearer understanding of these interacting factors will support the strategic selection of edible insect species and the optimization of rearing, harvesting, and processing practices.

### 4.6 Food safety considerations and future research perspectives

The microbiological safety of edible insects is an important aspect that complements their nutritional evaluation, particularly for wild-harvested species. Garofalo et al. (83) demonstrated considerable bacterial diversity in commercially processed crickets, locusts, and mealworms, including genera associated with spoilage and opportunistic infections. Caparros Megido et al. (84) reported high microbial loads in untreated fresh and traditionally smoked insects and showed that blanching, freeze-drying, and sterilization substantially reduced microbial counts, although bacterial spores were relatively resistant. Similarly, Fasolato et al. (85) observed considerable variation in microbial loads among processed edible insects and identified *Bacillus cereus* and related *Bacillus* species, emphasizing the importance of appropriate processing and hygienic handling. More recently, Pal et al. (86) reported diverse bacterial communities in commercially processed insect products, with *Bacillus* predominating and some *B. cereus* group isolates carrying toxin-associated genes. In the present study, the absence of microbiological assessment represents an important limitation; however, the nutritional and biochemical characterization provides a valuable baseline for subsequent food-safety investigations. Future studies integrating microbial profiling with the nutritional data generated here can help establish the safety of these wild-harvested insects, identify potential sources of contamination, and optimize processing, handling, and storage practices. This integrated approach would strengthen the scientific basis for their safe, sustainable, and nutritionally valuable utilization as alternative food resources.

## 5 Conclusion

This study provides a comprehensive evaluation of the nutritional composition and bioactive characteristics of 13 wild edible insect species, demonstrating considerable interspecific variation in proximate composition, amino acid profiles, fatty acid composition, phenolic and antioxidant content. Multivariate analyses further confirmed distinct species-specific nutritional patterns, with *P. barthelemyi* larvae exhibiting a superior amino acid profile and favorable unsaturated fatty acid composition, whereas *O. smaragdina* and *O. fuscidentalis* were characterized by comparatively higher phenolic and antioxidant contents. These findings demonstrate that the nutritional and functional attributes of edible insects are highly species-dependent and should not be generalized across taxa.

The results highlight the potential of selected edible insect species as nutrient-dense foods and promising sources of bioactive compounds for functional food and nutraceutical applications. However, their broader utilization requires standardized compositional databases, harmonized analytical methodologies, and further investigations into nutrient bioavailability, safety, and health benefits. Future research should also focus on optimizing rearing practices, dietary manipulation, and processing technologies to improve nutritional quality and product consistency while addressing consumer acceptance and regulatory requirements. Such integrated efforts will facilitate the sustainable incorporation of edible insects into future food systems and support their development as environmentally sustainable alternative protein sources.

## Data Availability

The original contributions presented in the study are included in the article/Supplementary material, further inquiries can be directed to the corresponding authors.
