# Supplementary material for: Nutritional diversity and bioactive potential of thirteen wild edible insects in India: implications for sustainable food and nutrition security
Source: Front Nutr. 2026 Oct 14;13:1942254. doi: 10.3389/fnut.2026.1942254 (PMC13713423; doi:10.3389/fnut.2026.1942254)
Supplement: Supplementary file 1 [file Image_1.pdf]

Supplementary Figure

Supplementary Figure 1

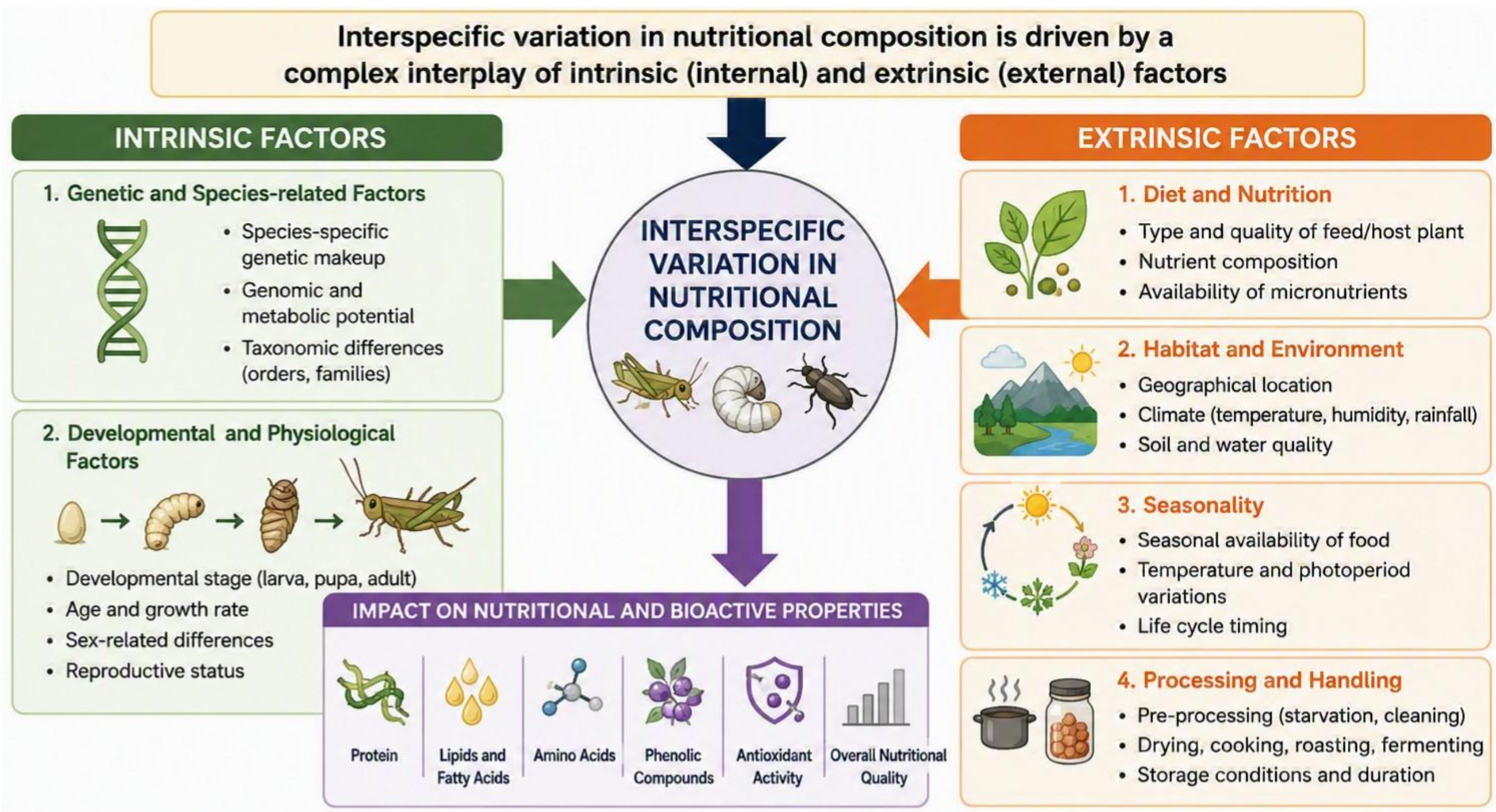

Supplementary Figure 1. Drivers of interspecific variation in the nutritional composition of edible insects.
