# Supplementary material for: Nutritional diversity and bioactive potential of thirteen wild edible insects in India: implications for sustainable food and nutrition security
Source: Front Nutr. 2026 Oct 14;13:1942254. doi: 10.3389/fnut.2026.1942254 (PMC13713423; doi:10.3389/fnut.2026.1942254)
Supplement: Supplementary file 2 [file Table_1.docx]

Supplementary Table

**Supplementary Table S1** Checklist of edible insect species documented from Nagaland, India, including their order and family, scientific names, developmental stage(s) consumed, and relevant literature sources.

| **Sl. No.** | **Order** | **Family** | **Scientific name** | **Edible stage** | **Mode of consumption** | **Reference(s)** |
| --- | --- | --- | --- | --- | --- | --- |
| 1. | Odonata | Libellulidae | *Diplacodes trivialis* | N | Boiled until cooked OR cooked with local ingredients | (1) |
| 2. |  |  | *Orthetrum pruinosum neglectum* | N |  |  |
| 3. |  |  | *Neurothemis fulvia* | N |  |  |
| 4. |  |  | *Crocothemis servilia servilia* | N |  |  |
| 5. |  |  | *Potamarcha congener* | N |  |  |
| 6. |  |  | *Orthetrum sabina sabina* | N |  |  |
| 7. |  |  | *Orthetrum triangulare triangulare* | N |  |  |
| 8. |  |  | *Pantala flavescens* | N |  |  |
| 9. | Orthoptera | Acrididae | *Acrida exaltata* | AD | Cooked with local ingredients |  |
| 10. |  |  | *Attractomorpha* sp. | AD |  |  |
| 11. |  |  | *Choroedocus robustus* | AD |  |  |
| 12. |  |  | *Chondracris rosea* | AD | Roasting over open fire OR toasting over low heat |  |
| 13. |  |  | *Gastrimargus africanus africanus* | AD | Cooked with local ingredients |  |
| 14. |  |  | *Hieroglyphus banian* | AD |  |  |
| 15. |  |  | *Melanoplus* sp. | AD | Roasting over open fire |  |
| 16. |  |  | *Melanoplus bivattus* | AD | Cooked with local ingredients |  |
| 17. |  |  | *Oxya hyla* | AD |  |  |
| 18. |  |  | *Oxya fuscovittata* | AD |  |  |
| 19. |  |  | *Phlaeoba infumata* | AD |  |  |
| 20. |  | Gryllidae | *Acheta domesticus* | AD |  |  |
| 21. |  |  | *Gryllus* sp. | AD |  |  |
| 22. |  |  | *Meloimorpha cincticornis* | AD |  |  |
| 23. |  |  | *Teleogryllus* sp. | AD |  |  |
| 24. |  |  | *Teleogryllus occipitalis* | AD |  |  |
| 25. |  |  | *Tarbinskiellus orientalis* | AD |  |  |
| 26. |  |  | *Tarbinskiellus protentosus* | AD | Cooked with local ingredients |  |
| 27. |  | Gryllotalpidae | *Gryllotalpa orientalis* | AD |  |  |
| 28. |  | Tettigoniidae | *Mecopoda nipponensis* | AD | Roasting over open fire OR toasting over low heat |  |
| 29. |  |  | *Mecopoda* *elongata* | AD |  |  |
| 30. |  |  | *Pseudophyllus titan* | AD |  |  |
| 31. |  |  | *Elimaea securigera* | AD | Cooked with local ingredients |  |
| 32. |  |  | *Tettigonia* sp. | AD |  |  |
| 33. |  | Schizodactylidae | *Schizodactylus monstrosus* | AD |  |  |
| 34. | Mantodea | Manitidae | *Tenodera sinensis* | AD | Roasting over open fire |  |
| 35. |  |  | *Heirodula coarctata* | AD |  |  |
| 36. | Blattodea | Termitidae | *Macrotermes* sp. | AD | Cooked with local ingredients |  |
| 37. | Hemiptera | Aetalionidae | *Darthula hardwickii* | N | Cooked with local ingredients OR dry-fried in oil |  |
| 38. |  | Belostomatidae | *Lethocerus indicus* | AD |  |  |
| 39. |  | Cicadidae | *Tibicen pruinosa* | AD |  |  |
| 40. |  |  | *Dundubia intemerata* | AD |  |  |
| 41. |  |  | *Dundubia oopaga* | AD |  |  |
| 42. |  |  | *Pomponia* sp. | AD |  |  |
| 43. |  |  | *Pycna repanda* | AD |  |  |
| 44. |  |  | *Cryptotympana corvus* | AD |  | ** |
| 45. |  | Coreidae | *Dalader planiventris* | AD |  | (1) |
| 46. |  |  | *Anoplocnemis phasiana* | AD |  |  |
| 47. |  |  | *Notobitus meleagris* | AD |  |  |
| 48. |  | Dinidoridae | *Aspongopus nepalensis* | AD |  |  |
| 49. |  |  | *Coridius janus* | AD | Cooked with local ingredients OR prepared as chutney |  |
| 50. |  |  | *Coridius chinensis* | AD |  |  |
| 51. |  |  | *Coridius singhalanus* | AD |  |  |
| 52. |  | Nepidae | *Laccotrephes ruber* | AD | Cooked with local ingredients OR dry-fried in oil |  |
| 53. |  | Pentatomidae | *Chinavia hilaris* | AD | Cooked with local ingredients OR prepared as chutney OR dry-fried in oil |  |
| 54. |  |  | *Cyclopelta siccifolia* | AD |  |  |
| 55. |  |  | *Dolycoris* sp. | AD |  |  |
| 56. |  |  | *Erthesina fullo* | AD |  |  |
| 57. |  |  | *Eurostus grossipes* | AD | Roasting over open fire |  |
| 58. |  |  | *Udonga montana* | AD | Cooked with local ingredients OR prepared as chutney OR dry-fried in oil |  |
| 59. |  | Tessaratomidae | *Tessaratoma javanica* | AD | Roasting over open fire |  |
| 60. |  |  | *Pycanum* sp. | AD | Roasting over open fire OR dry-fried in oil | (2) |
| 61. |  |  | Plant hopper | AD | Prepared as chutney OR dry-fried in oil | ** |
| 62. |  | Naucoridae | *Gestroiella insignis* | AD | Boiled until cooked OR cooked with local ingredients OR dry-fried in oil | (3) |
| 63. | Coleoptera | Cerambycidae | *Aeolesthes holosericea* | L, P | Roasted over open fire OR dry-fried in oil | (4) |
| 64. |  |  | *Anoplophora* sp. | A | Cooked with local ingredients OR roasted over open fire OR dry-fried in oil | (1) |
| 65. |  |  | *Batocera rubus* | L, P |  |  |
| 66. |  |  | *Batocera parryi* | L, P |  |  |
| 67. |  |  | *Batocera rufomaculata* | L, P |  |  |
| 68. |  |  | *Orthosoma brunneum* | A |  |  |
| 69. |  |  | *Thysia wallichi* | L, P |  | (5) |
| 70. |  |  | **Apriona germari* | L, P |  | (6) |
| 71. |  |  | **Neoplocaederus obesus* | A |  |  |
| 72. |  | Chrysomelidae | *Aplosonyx chalybaeus* | L |  | (1) |
| 73. |  | Curcurlionidae | *Rhynchophorus ferrigineus* | A |  |  |
| 74. |  | Dytiscidae | *Cybister limbatus* | AD | Cooked with local ingredients |  |
| 75. |  |  | *Cybister tripunctatus lateralis* | AD |  |  |
| 76. |  |  | *Sandracottus manipurensis* | AD |  |  |
| 77. |  | Hydropillidae | *Hydrophilus caschmirensis* | AD |  |  |
| 78. |  | Scarabaeidae | *Xylotrupes gideon* | AD | Roasted over open fire OR dry-fried in oil |  |
| 79. |  |  | *Lepidiota stigma* | AD |  |  |
| 80. |  |  | *Holotricha* sp. | AD |  |  |
| 81. | Hymenoptera | Apidae | *Apis cerana indica* | A | Cooked with local ingredients OR eaten raw (honey) |  |
| 82. |  |  | *Apis dorsata dorsata* | A |  |  |
| 83. |  |  | *Apis dorsata laboriosa* | A |  |  |
| 84. |  |  | *Apis florea* | A |  |  |
| 85. |  |  | *Lepidotrigona arcifera* | A | Raw (honey) |  |
| 86. |  |  | *Lepidotrigona ventralis* | A |  | (3) |
| 87. |  |  | *Lophotrigona canifrons* | A | Raw (honey) | (1,7,8,9) |
| 88. |  |  | *Tetragonula iridipennis* | A |  | (3) |
| 89. |  |  | **Tetragonula laeviceps* | A |  | (7,8,9) |
| 90. |  |  | **Tetragonula gressitti* | A |  | (10) |
| 91. |  |  | *Xylocopa violacea* | H |  | (1) |
| 92. |  | Formicidae | *Oecophylla smaragdina* | A | Boiled until cooked OR cooked with local ingredients OR prepared as chutney |  |
| 93. |  | Vespidae | *Parapolybia varia* | A | Boiled until cooked OR cooked with local ingredients OR dry-fried in oil |  |
| 94. |  |  | *Polistes olivaceus* | A |  |  |
| 95. |  |  | *Polistes stigma* | A |  |  |
| 96. |  |  | *Polistes* sp. | A |  |  |
| 97. |  |  | *Provespa barthelemyi* | A |  |  |
| 98. |  |  | *Ropalida rufoplagiata* | A |  |  |
| 99. |  |  | *Ropalida* sp. | A |  |  |
| 100. |  |  | *Vespa affinis indosinensis* | A |  |  |
| 101. |  |  | *Vespa auraria* | A |  |  |
| 102. |  |  | *Vespa basalis* | A |  |  |
| 103. |  |  | *Vespa bicolor* | A |  |  |
| 104. |  |  | *Vespa ducalis* | A |  |  |
| 105. |  |  | *Vespa mandarinia* | A |  |  |
| 106. |  |  | *Vespa tropica tropica* | A |  |  |
| 107. |  |  | *Vespa* sp. | A |  | ** |
| 108. |  |  | *Vespa soror* | A |  | (1) |
| 109. |  |  | *Vespula orbata* | A |  |  |
| 110. |  |  | *Vespula* sp. | A |  |  |
| 111. | Lepidoptera | Cossidae | *Cossus* sp. | L, P |  |  |
| 112. |  | Crambidae | *Omphisa fuscidentalis* | L, P | Boiled until cooked OR cooked with local ingredients OR dry-fried in oil OR steamed |  |
| 113. |  | Hesperiidae | *Erionata torus* | L, P | Boiled until cooked OR cooked with local ingredients OR dry-fried in oil |  |
| 114. |  | Lasiocampidae | *Malacosoma* sp. | L, P |  |  |
| 115. |  | Noctuidae | *Pericyma cruegeri* | L |  |  |
| 116. |  | Saturniidae | *Antheraea assamensis* | L |  |  |
| 117. |  |  | *Antheraea mylitta* | L |  |  |
| 118. |  |  | *Samia cynthia ricini* | L, P | Boiled until cooked OR cooked with local ingredients OR dry-fried in oil | (1) |
| 119. |  | Entromidae | *Andraca* sp. | L, P |  | (11) |
| 120. | Diptera | Tipulidae | *Tipula* sp. | L |  | (1) |
| 121. | Megaloptera |  | Unidentified | L |  | (2) |
| 122. | Plecoptera |  | Unidentified | N |  |  |

*Edible stage: A-All; AD-Adult; H- Honey; L-Larva, N-Nymph; P-Pupa*

** Species presence reported. New addition to the edible insect list of Nagaland*

*** New addition to the edible insect list of Nagaland*

**References**

1. Mozhui L, Kakati LN, Kiewhuo P, Changkija S. Traditional knowledge of the utilization of edible insects in Nagaland, North-East India. *Foods*. (2020a) 9(7):852. https://doi.org/10.3390/foods9070852
2. Chang YB, Chhetri RK, Mozhui L. Indigenous knowledge on edible insect consumption of the Chang tribe, Tuensang, Nagaland. In: Khalid MA, Kumar S, Yadav E, Pankaj PP, Kharwar RK, editors. Vanishing biodiversity: Global threats to flora and fauna*.* Academic Publishers & Distributors, Lucknow, UP, India (2025). p. 487–501. https://doi.org/10.5281/zenodo
3. Namsong N, Chhetri RK, Mozhui L. “Entomophagy practices of the Phom tribe of Longleng district, Nagaland, India”. In: Khalid MA, Kumar S, Yadav E, Pankaj PP, Kharwar RK, editors. Vanishing biodiversity: Global threats to flora and fauna*.* Academic Publishers & Distributors, Lucknow, UP, India, Academic Publishers & Distributors (2025). p. 475– 486. https://doi.org/10.5281/zenodo
4. Pongener A, Bendang A, Yenisetti SC. An assessment of the nutritional quality and future prospects of *Aplosonyx chalybaeus* and *Aeolesthes holosericea* (Coleoptera) in Nagaland, India. *IJSR*. (2019) 8:550–556. https://dx.doi.org/10.21275/ART20196016
5. Kiewhuo P, Mozhui L, Kakati LN, Lirikum, Meyer- Rochow VB. Traditional rearing techniques of the edible Asian giant hornet (*Vespa mandarinia* Smith) and its socio-economic perspective in Nagaland, India. *J Insects Food Feed*. (2022) 8(3):325–336. https://doi.org/10.3920/JIFF2021.0088
6. Mozhui L, Rana A, Neikha K, Kakati LN. A checklist of long horn beetles (Coleoptera: Cerambycidae) of Lumami, Zunheboto District, Nagaland with 23 new records. *Halteres*. (2020b) 11:118–128. doi: 10.5281/zenodo.4405843
7. Singh AK. Traditional meliponiculture by Naga tribes in Nagaland, India. *ITJK*. (2016) 15(4):693–699.
8. Chauhan A, Singh HK. Nest architecture of stingless bees *Tetragonula iridipennis* and *Lepidotrigona ventralis* in Nagaland. *Indian J Entomol*. (2019) 81(3):613–617. doi: 10.5958/0974-8172.2019.00127.5
9. Chauhan A, Singh HS. Mapping of stingless bee fauna in Nagaland. *Int J Farm Sci*. (2022) 12(2):117–122. doi: 10.5958/2250-0499.2022.00061.1
10. Chauhan A, Singh HK. A new record of stingless bee, *Tetragonula gressitti* Sakagami (Hymenoptera: Apidae: Meliponini) from Nagaland, India. *Int J Farm Sci*. (2020) 10(3and4):83–87. doi: 10.5958/2250-0499.2020.00057.9
11. Pongener A. (2022) Diversity and nutritional status of certain edible insects in Nagaland. [PhD thesis]. [Department of Zoology: Nagaland University]. Lumami, Nagaland, India.
