# Supplementary material for: Nutritional diversity and bioactive potential of thirteen wild edible insects in India: implications for sustainable food and nutrition security
Source: Front Nutr. 2026 Oct 14;13:1942254. doi: 10.3389/fnut.2026.1942254 (PMC13713423; doi:10.3389/fnut.2026.1942254)
Supplement: Supplementary file 3 [file Table_2.docx]

Supplementary Table

**Supplementary Table S2** Traditional medicinal uses of edible insects in Nagaland, India. **Adapted from Mozhui et al. (1) with additional information incorporated by the authors.**

| **Sl. No.** | **Order** | **Family** | **Scientific name/Insect order** | **Parts used** | **Disease treated/traditional medicinal use/other uses** |
| --- | --- | --- | --- | --- | --- |
| 1. | Odonata | Libellulidae | *Crocothemis servilia servilia* | N | Dermatological, respiratory, musculoskeletal, ophthalmological |
| 2. |  |  | *Diplacodes trivialis* |  |  |
| 3. |  |  | *Neurothemis fulvia* |  |  |
| 4. |  |  | *Orthetrum pruinosum neglectum* |  |  |
| 5. |  |  | *Orthetrum sabina sabina* |  |  |
| 6. |  |  | *Orthetrum triangulare triangulare* |  |  |
| 7. |  |  | *Pantala flavescens* |  |  |
| 8. |  |  | *Potamarcha congenera* |  |  |
| 9. | Orthoptera | Acrididae | *Melanoplus* sp*.* | AD | Gastrointestinal, diabetes |
| 10. |  | Gryllidae | *Gryllus* sp*.* | AD | Gastrointestinal |
| 11. |  |  | *Tarbinskiellus protentosus* | AD | Gastrointestinal, malaria |
| 12. |  | Tettigoniidae | *Pseudophyllus titan* | AD | Fever |
| 13. |  |  | *Elimaea scurigera* | AD | Nutritional supplementation |
| 14. | Mantodea | Manitidae | *Tenodera sinensis* | AD | Dermatological |
| 15. |  |  | *Heirodula coarctata* | AD | Urological |
| 16. | Blattodea | Blaberidae | *Epilampra* sp. | AD | Gastrointestinal, appetite stimulation |
| 17. |  | Termitidae | *Macrotermes* sp. | A | Nutritional supplementation |
| 18. | Phasmatodea | Lonchoidae | *Carausius* sp*.* | AD | Dermatological |
| 19. | Hemiptera | Aetalionidae | *Darthula hardwickii* | N | Musculoskeletal, jaundice |
| 20. |  | Belostomatidae | *Lethocerus indicus* | AD | Gastrointestinal, Dermatological, respiratory, musculoskeletal, cardiovascular |
| 21. |  | Coreidae | *Notobitus meleagris* | AD | Gastrointestinal |
| 22. |  | Dinidoridae | *Aspongopus nepalensis* | AD | Jaundice |
| 23. |  |  | *Coridius singhalanus* | AD | Lactation promotion (galactagogue), jaundice, malaria |
| 24. |  | Nepidae | *Laccotrephes ruber* | AD | Gastrointestinal, dermatological, cardiovascular |
| 25. |  | Pentatomidae | *Udonga montana* | AD | Dermatological, respiratory, pain, diabetes |
| 26. |  | Naucoridae | **Gestroiella insignis* | AD | Gastrointestinal |
| 27. | Neuroptera | Myrmeleontidae | *Myrmeleon* sp*.* | AD | Dermatological |
| 28. | Coleoptera | Dytiscidae | *Cybister limbatus* | AD | Gastrointestinal |
| 29. |  |  | *Cybister tripunctatus lateralis* | AD | Gastrointestinal |
| 30. |  | Hydrophillidae | *Hydrophilus caschmirensis* | AD | Dermatological |
| 31. |  | Meloidae | *Mylabris variabilis* | AD | Dermatological |
| 32. |  | Cerambycidae | **Aeolesthes holosericea* | L, P | Gastrointestinal, aphrodisiac, pain, malaria, typhoid, musculoskeletal |
| 33. |  |  | *Batocera rubus* | L |  |
| 34. |  |  | *Batocera parryi* |  |  |
| 35. |  |  | *Batocera rufomaculata* |  |  |
| 36. |  |  | *Orthosoma brunneum* | L | Gastrointestinal, respiratory, aphrodisiac, typhoid, malaria |
| 37. |  |  | **Neoplocaederus obesus* | A |  |
| 38. | Hymenoptera | Apidae | *Apis cerana indica* | A, BC, BW, H, L, P | Gastrointestinal, dermatological, respiratory, cardiovascular, pain, cholera, musculoskeletal, ophthalmological, common ailments, hangovers, vertigo, nutritional supplementation |
| 39. |  |  | *Apis dorsata dorsata* | A, BC, H, L, P | Gastrointestinal, respiratory, cardiovascular, appetite stimulation |
| 40. |  |  | *Apis dorsata laboriosa* | BC, H, L, P | Gastrointestinal, respiratory, common ailments |
| 41. |  |  | *Apis florea* | BC, H, L, P |  |
| 42. |  |  | *Lepidotrigona arcifera* | H, NE | Gastrointestinal, dermatological, respiratory, labour facilitation, cardiovascular, pain, musculoskeletal, poisonous animal bites, oncological, cultural and ritual practices, ophthalmological, toothaches |
| 43. |  |  | **Lepidotrigona ventralis* |  |  |
| 44. |  |  | *Lophotrigona canifrons* |  |  |
| 45. |  |  | **Tetragonula iridipennis* |  |  |
| 46. |  |  | **Tetragonula laeviceps* |  |  |
| 47. |  |  | **Tetragonula gressitti* |  |  |
| 48. |  | Formicidae | *Oecophylla smaragdina* | A | Respiratory, pain, typhoid, malaria, oedema |
| 49. |  | Vespidae | *Provespa barthelemyi* | A | Musculoskeletal, insomnia |
| 50. |  |  | *Vespa mandarina* | A | Diabetes, nutritional supplementation |
| 51. |  |  | *Vespa tropica tropica* | A | Nutritional supplementation |
| 52. | Lepidoptera | Cossidae | *Cossus* sp*.* | L | Gastrointestinal, respiratory, cardiovascular, pain, typhoid, musculoskeletal, diabetes, general weakness, fatigue |
| 53. |  | Crambidae | *Omphisa fuscidentalis* | L | Musculoskeletal |
| 54. |  | Hesperiidae | *Erionata torus* | L | Aphrodisiac, ophthalmological |
| 55. |  | Lasiocampidae | *Malacosoma* sp*.* | L | Musculoskeletal |
| 56. |  | Saturniidae | *Samia cynthia ricini* | L | Pain, cardiovascular, diabetes, and nutritional supplementation |
| 57. | Diptera | Tipulidae | *Tipula* sp*.* | L | Pain, measles, ophthalmological, fatigue, physical exhaustion |
| 58. | *Megaloptera |  |  | L | Pain, fatigue, physical exhaustion |
| 59. | *Plecoptera |  |  | N | Pain, fatigue, physical exhaustion |

*Parts used: A-All; AD-Adult; BC- Bee Comb; H- Honey; L-Larva, N-Nymph; NE-Nest Entrance; P-Pupa; BW-Bee Wax*

**New addition to the medicinal insect list of Nagaland*

**References**

1. Mozhui L, Kakati LN, Meyer-Rochow VB. Entomotherapy: a study of medicinal insects of seven ethnic groups in Nagaland, North-East India. *J Ethnobiol Ethnomed*.  (2021) 17(1):17. https://doi.org/10.1186/s13002-021-00444-1
